# Integrating ayurvedic eye care into primary health practice: an exploratory study on the combined effect of *pratimarsha nasya*, *avagundana*, and *aschyotana* in meibomian gland dysfunction

**DOI:** 10.3389/fmed.2025.1732091

**Published:** 2026-01-21

**Authors:** Sreelekha P, Sushma Naranappa Salethoor, Shanti K

**Affiliations:** Amrita Vishwa Vidyapeetham Amrita School of Ayurveda, Kollam, India

**Keywords:** ayurveda, ayurvedic ophthalmology, dry eye disease, integrative medicine, Meibomian Gland Dysfunction, *pratimarsha nasya*, primary care, *shigrupallava arka*

## Abstract

**Background:**

Meibomian Gland Dysfunction (MGD) has become increasingly common in community practice, often presenting as dryness, irritation, and ocular fatigue. Factors such as prolonged screen exposure, environmental irritants, and advancing age contribute to its growing burden in primary health settings. Conventional management usually focuses on symptom relief, leaving a need for safer, sustainable options that can be applied easily in routine care. Ayurvedic eye therapies, known for their gentle yet restorative effects, may offer such an alternative.

**Methods:**

A single-arm, open-label clinical study was conducted on 30 patients with clinically diagnosed Meibomian Gland Dysfunction (MGD). The treatment protocol consisted of daily nasal oil instillation (*pratimarsha nasya*) using Anu oil for 30 days, along with medicated eye drops (*aschyotana*) prepared from *Moringa oleifera* leaves and localized warm ocular fomentation (*avagundana*) using a herbal bolus immersed in a Triphala-based decoction for the first 15 days. Clinical outcomes were assessed using both subjective and objective parameters, including the Ocular Surface Disease Index (OSDI), Tear Film Break-Up Time (TBUT), Schirmer’s Test I, fluorescein staining, and meibomian gland expressibility. Assessments were performed at baseline, immediately after treatment, and during follow-up visits. Data were analyzed using the Friedman test and Wilcoxon Signed-Rank test.

**Results:**

Patients showed marked improvement in all clinical parameters. OSDI, TBUT, and Schirmer’s scores improved significantly (*p* < 0.001). Fluorescein staining was reduced to nil after treatment and remained stable during follow-ups, while meibomian gland expressibility improved consistently, reflecting better tear film stability and glandular function. No side effects or adverse reactions were reported.

**Conclusion:**

This exploratory study suggests that a simple Ayurvedic care regimen may be associated with improvement in symptoms and ocular surface parameters in individuals with Meibomian Gland Dysfunction. Given its non-invasive nature, ease of administration, and suitability for low-resource settings, this approach may have potential relevance as a supportive strategy within primary eye care. However, these findings are preliminary, and further controlled studies are required to confirm effectiveness and define its role in community-level preventive and promotive eye health.

**Limitations of the study:**

This was a single-arm exploratory study with a small sample size and limited follow-up. Consequently, the findings are preliminary and require confirmation in controlled trials.

## Introduction

Meibomian Gland Dysfunction (MGD) represents a chronic, diffuse disorder of the meibomian glands, primarily marked by ductal obstruction or by changes in the composition and quantity of glandular secretions. These alterations compromise the stability of the tear film, resulting in ocular irritation, inflammation, and surface discomfort. A range of intrinsic and extrinsic factors including advancing age, hormonal influences, systemic medications, and lifestyle patterns are known to contribute to the onset and progression of this condition. A reduced lipid layer causes excessive evaporation of tears, resulting in hyperosmolarity and instability of the tear film (1). Consequently, patients experience ocular discomfort, redness, inflammation, and a sensation of grittiness or a foreign body in the eye. Although the exact pathophysiology of MGD is not fully understood, it is associated with poor gland expressibility, altered meibum quality, lid margin inflammation, and, in some cases, irreversible gland atrophy or dropout (2).

MGD is one of the most common conditions encountered in ophthalmic practice and remains the leading cause of evaporative dry eye disease. According to Amano, the prevalence of MGD in Asian populations ranges between 46.2 and 68.0% (3). In India, a hospital-based study reported a prevalence of around 55%, while community-based studies show rates of approximately 30%. A population-based study from South India by Singh et al. found MGD to be the most common cause of dry eye symptoms, seen in 100 (40%) patients, followed by chronic allergic conjunctivitis in 75 (30%) patients (4). With its rising global prevalence across age groups, MGD poses a significant public health challenge. Primary care physicians (PCPs), often the first point of contact for DED patients, require timely screening and management strategies (5).

Currently, there is no gold-standard treatment for MGD. Management typically includes a combination of conservative measures such as warm compresses and lid hygiene, along with medical treatments like antibiotics, and nonsteroidal or steroidal anti-inflammatory agents. Procedural options such as intraductal meibomian gland probing, electronic heating devices, intense pulsed light therapy, and intranasal neurostimulation are also being explored (2). However, data from multiple placebo-controlled trials and meta-analyses have raised concerns about the adverse effects of long-term NSAID use on ocular, gastrointestinal, cardiovascular, hepatic, renal, and even pulmonary systems (6).

Given these challenges, there is a growing need for safe, sustainable, and holistic alternatives that can be integrated into primary eye care and general practice. Traditional Ayurvedic medicine offers a thoughtful, individualized way of dealing with conditions like MGD. Instead of focusing only on immediate relief, it works to correct underlying imbalances by harmonizing the *doshas* and clearing blockages in the channels of the eyes. This approach not only improves symptoms naturally but may also reduce the long-term use of antibiotics and steroids.

The clinical features of Meibomian Gland Dysfunction show parallels with classical Ayurvedic descriptions of inflammatory (*kaphaja abhisjyanda*) and dryness-predominant eye conditions (*shushkakshipaka*) associated with impaired lubrication and obstruction. Accordingly, the treatment strategy was designed to address glandular obstruction and surface dryness in the early phase, followed by measures aimed at restoring ocular lubrication and functional balance in later stages. Topical eye instillation and localized warm ocular fomentation were used during the acute phase to relieve obstruction and inflammation, while daily nasal oil instillation was employed as a supportive therapy to improve mucosal hydration, circulation, and functional regulation of ocular tissues.

Medicated eye drop instillation (*aschyotana*) is traditionally considered a primary intervention in acute ocular conditions, as it allows direct delivery of therapeutic agents to the ocular surface. Localized warm ocular fomentation (*avagundana*) is employed in the early stage of disease (*ama avstha*), where gentle heat combined with herbal decoctions helps soften secretions and facilitate the expression of obstructed meibomian gland contents. Daily nasal oil instillation (*pratimarsha nasya*) serves as a supportive measure by promoting mucosal lubrication, improving regional circulation, and contributing to overall functional regulation of ocular tissues.

*Moringa oleifera* (*sigru*) is traditionally recognized for its beneficial effects on ocular health and its anti-inflammatory activity, which is supported by experimental evidence (7). The distillate preparation (*arka*) derived from tender *Moringa oleifera* leaves offers practical advantages, including improved compatibility, longer shelf life, and enhanced bioavailability. As a low-residue, rapidly absorbable formulation, the distillate allows quicker tissue penetration and systemic distribution, supporting its use in ocular applications that require prompt therapeutic action.

Although many clinical experiences and traditional records indicate that Ayurvedic treatments can relieve the symptoms of Meibomian Gland Dysfunction, there is still a shortage of systematic studies using measurable clinical outcomes. Bringing such time-tested practices into the setting of primary eye care may offer a practical, safe, and affordable complement to existing therapies. With this idea in mind, the present exploratory study was designed to evaluate the combined use of *pratimarsha nasya*, *avagundana*, and *aschyotana* in managing MGD, while also highlighting how Ayurveda-based eye care could be meaningfully integrated into routine primary health services.

## Materials and methods

Patients reporting to the Outpatient and Inpatient Departments of Śālākya Tantra were screened, and 30 individuals who met the inclusion and exclusion criteria were enrolled in the study. Participants of either gender, aged between 20 and 70 years, presenting with clinical signs and symptoms of Meibomian Gland Dysfunction (MGD) were included (4). Patients undergoing long-term treatment with systemic medications, immunosuppressants, or topical steroids, as well as pregnant and lactating women, were excluded from the study.

Written informed consent, in accordance with the principles of the Declaration of Helsinki, was obtained from all participants in their native language after a detailed explanation of the study protocol. The study protocol received ethical clearance from the Institutional Ethics Committee (Approval No: IEC. ASA.PGR.04/24) and was registered with the Clinical Trial Registry of India (CTRI/2024/07/071440).

This was a single-arm, open-labeled exploratory clinical study involving 30 participants. As this was an exploratory feasibility study, no formal sample size calculation was performed. A sample size of 30 participants was chosen based on commonly recommended numbers for pilot studies (*n* = 20–40), which are adequate for estimating effect sizes and variability for designing future randomized controlled trials. The intervention consisted of daily nasal oil instillation using Anu oil for 30 days, combined with medicated eye drop instillation prepared from *Moringa oleifera* leaf distillate and localized warm ocular fomentation using a herbal poultice immersed in a Triphala-based decoction for the initial 15 days.

Assessment was based on both subjective and objective criteria and was done by a single trained examiner. Subjective evaluation was carried out using the Ocular Surface Disease Index (OSDI) Questionnaire (8), while objective parameters included Tear Film Break-Up Time (TBUT), Schirmer Test I, Fluorescein Staining, photographic assessment of meibomian duct obstruction and gland expressibility (9). Fluorescein staining of the ocular surface was evaluated using the Oxford grading scale. A single trained ophthalmologist performed all assessments to maintain consistency; examiner blinding was not feasible in this single-arm study. The instillation technique, observation interval, and slit-lamp illumination settings were standardized across all visits. These subjective and objective parameters were recorded before and after treatment and reassessed during two follow-up visits conducted fortnightly over the subsequent month.

The primary outcome measure was the change in Ocular Surface Disease Index (OSDI) score from baseline to post-treatment. TBUT, Schirmer’s test, fluorescein staining and gland expressibility were defined as secondary exploratory outcomes.

The statistical significance of changes in mean scores before and after treatment was analyzed using the Friedman Test, and the Wilcoxon Signed-Rank Test was applied to evaluate variations in subjective and objective scores across six pairs of four different time points. All statistical analyses were performed using SPSS software for Windows (see Table 1).

**Table 1:** Therapeutic intervention adopted in this study.

Intervention	Formulation used	Frequency of administration	Dose/duration per session	Total duration of treatment	Follow-up schedule
*Pratimarsha Nasya* (nasal oil instillation)	*Anutaila*	Twice daily (6.30 a.m. and 6 p.m.)	2 bindu (≈1 mL) each time	Day 01 to Day 30 (30 days)	First follow-up—Day 46. Second follow-up—Day 61.
*Aschyotana* (medicated eye drops)	*Sigrupallava arka*	Twice daily (8 a.m. and 5 p.m.)	8 bindu (≈4 mL) each time	Day 01 to Day 15 (15 days)	
*Avagundana* (localized warm fomentation)	*Sigru Pallava Choorna* in *Triphala Kashaya*	Twice daily (10 a.m. and 4 p.m.)	10 min per session	Day 01 to Day 15 (15 days)	

The physicochemical analysis of the *Moringa oleifera* leaf distillate, Anu oil, and Triphala powder was conducted to ensure authenticity, purity, and quality prior to clinical use. Standard analytical parameters, including appearance, pH, specific gravity, refractive index, and loss on drying, were evaluated in accordance with established pharmacopoeial methods.

Sterility testing of the *Moringa oleifera* leaf distillate, Anu oil, Triphala powder, and the herbal poultice prepared from *Moringa oleifera* leaves was carried out at the Centre for Advanced Research in Ayurveda of the institution. All preparations were evaluated for microbial contamination using standard sterility testing procedures to confirm their safety for ophthalmic and nasal use.

## Results

Statistical analysis was performed using IBM SPSS Statistics Version 26. A total of 33 patients were registered for the study, of which 3 patients were excluded due to loss to follow-up. Thus, data from 30 participants who completed the full course of treatment were included in the final analysis.

The demographic distribution of patients was analyzed based on age, sex, occupation, dietary habits, *agni* (digestive strength), socioeconomic status, and prakriti (constitution). It was observed that the majority of participants (73.3%) belonged to the 20–39 years age group, indicating a higher prevalence of Meibomian Gland Dysfunction among younger adults in this cohort.

### Results on OSDI questionnaire

**Figure 1:**
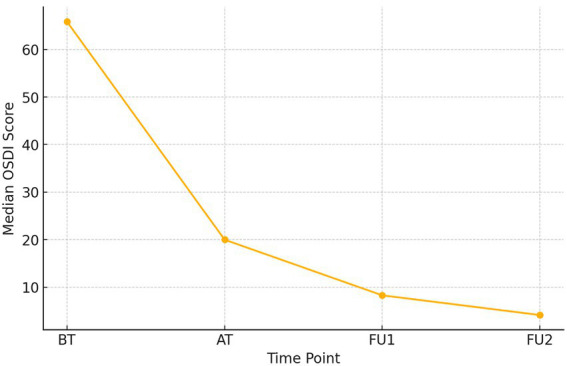
The line graph provides the median OSDI values across the treatment time points (BT, Before treatment; AT, After treatment; FU1, Follow-up 1; FU2, Follow-up 2).

Because the OSDI scores were not normally distributed, results are presented as medians with interquartile ranges (IQR). A progressive reduction in symptom severity was observed across all four time points: baseline 65.85 (IQR 62.50–72.50), after treatment 20.00 (16.60–22.10), first follow-up 8.30 (8.30–10.98), and second follow-up 4.16 (4.10–4.90). A Friedman test demonstrated a statistically significant difference in OSDI scores across the treatment timeline, χ^2^(3) = 90.00, *p* = 2.19 × 10^−19^. Post-hoc Wilcoxon Signed-Rank Tests showed significant pairwise improvements between all time points (all *p* < 0.001, Bonferroni-adjusted). These results reflect a clinically meaningful reduction in dry eye symptoms, maintained through the follow-up period (see Figure 1).

### Results on tear film break-up time (TBUT)

TBUT values improved markedly over the study period in both eyes. Because the TBUT data did not follow normal distribution, results are expressed as median with interquartile range (IQR). In the right eye, TBUT increased from 2.0 (2.0–3.0) at baseline to 9.0 (8.0–10.0) after treatment, 11.0 (11.0–11.0) at the first follow-up and 11.0 (11.0–12.0) at the second follow-up. Similarly, the left eye improved from 3.0 (2.0–3.75) at baseline to 10.0 (9.0–11.0) after treatment, 11.5 (11.0–12.0) at follow-up 1 and 12.0 (11.0–12.75) at follow-up 2.

**Figure 2:**
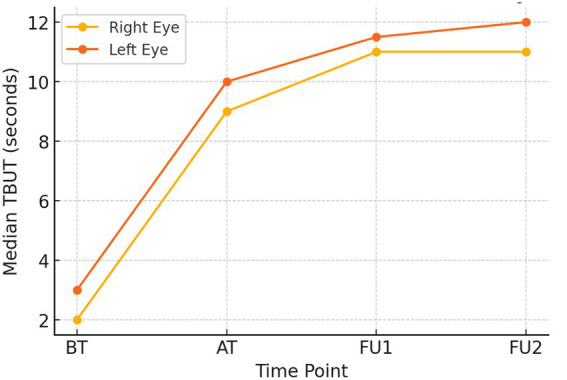
The line graph provides the median TBUT (in seconds) values of both eyes across the treatment time points (BT, Before treatment; AT, After treatment; FU1, Follow-up 1; FU2, Follow-up 2).

A Friedman test demonstrated a difference across the four time points in both eyes (right eye χ^2^(3) = 85.332, *p* < 0.001; left eye χ^2^(3) = 83.700, *p* < 0.001). Post-hoc Wilcoxon Signed-Rank Tests showed pairwise improvements between all time points in both eyes (all *p* < 0.001). These results indicate a consistent and clinically meaningful enhancement in tear film stability throughout the treatment and follow-up period (see Figure 2).

### Results on Schirmer’s test

Because the Schirmer’s Test I values did not follow a normal distribution, results are expressed as median with interquartile range (IQR). A progressive increase in tear secretion was observed in both eyes throughout the intervention period. In the right eye, median ST-I increased from 2.0 (1.25–3.0) at baseline to 11.0 (10.0–11.0) after treatment, 13.0 (13.0–14.75) at the first follow-up and 15.0 (13.0–15.0) at the second follow-up. Similarly, the left eye improved from 3.0 (2.0–4.0) at baseline to 12.0 (11.0–13.0) after treatment, 15.0 (13.25–15.0) at follow-up 1, and 15.0 (15.0–15.75) at follow-up 2.

**Figure 3:**
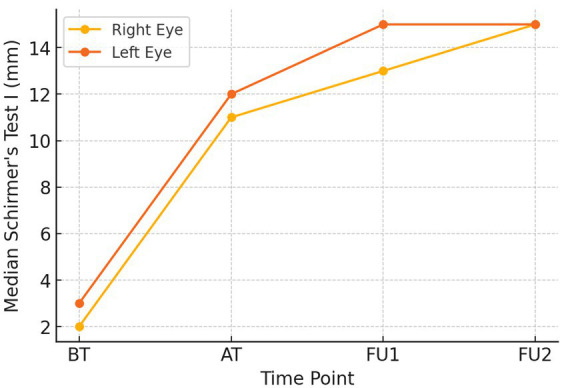
The line graph provides the median Schirmer test score (in mm) values of both eyes across the treatment time points (BT, before treatment; AT, After treatment; FU1, Follow-up 1; FU2, Follow-up 2).

A Friedman test confirmed a statistically significant difference in Schirmer’s values across the four time points in both eyes (right eye χ^2^ (3) = 83.37, *p* = 5.81 × 10^−18^; left eye χ^2^(3) = 84.52, *p* = 3.29 × 10^−18^). Pairwise Wilcoxon Signed-Rank tests demonstrated improvements between each assessment interval for both eyes (all *p* < 0.001). Taken together, these findings indicate a sustained and clinically meaningful enhancement in aqueous tear production and ocular surface hydration following treatment (see Figure 3).

### Results on fluorescein staining

Fluorescein staining was graded using the Oxford grading scale (0–5). In this cohort, observed scores ranged from grade 0 to grade 2 at baseline and reported using median and interquartile range (IQR). At baseline, participants showed evidence of ocular surface disturbance, with median staining scores of 2.0 (IQR 2.0–2.0) in the right eye and 2.0 (IQR 2.0–2.0) in the left eye. After treatment, median scores reduced to 0 (IQR 0–0) in both eyes and remained unchanged at the two follow-up visits.

As all eyes demonstrated complete resolution of staining from the immediate post-treatment assessment onwards, there was no variability in the post-treatment data; therefore, statistical comparison tests were not applicable. The uniform reduction to zero and maintenance of this outcome during follow-up suggest rapid epithelial recovery and sustained improvement in ocular surface integrity.

### Results on meibomian gland expressibility

Meibomian gland expressibility was evaluated using a 1–3 grading scale. Since the values were ordinal and non-normally distributed, the results are presented as median and interquartile range (IQR). At baseline, expressibility scores indicated varying degrees of obstruction, with medians of 2.0 (IQR 1.0–2.0) in the right eye and 2.0 (IQR 2.0–2.0) in the left eye.

**Figure 4:**
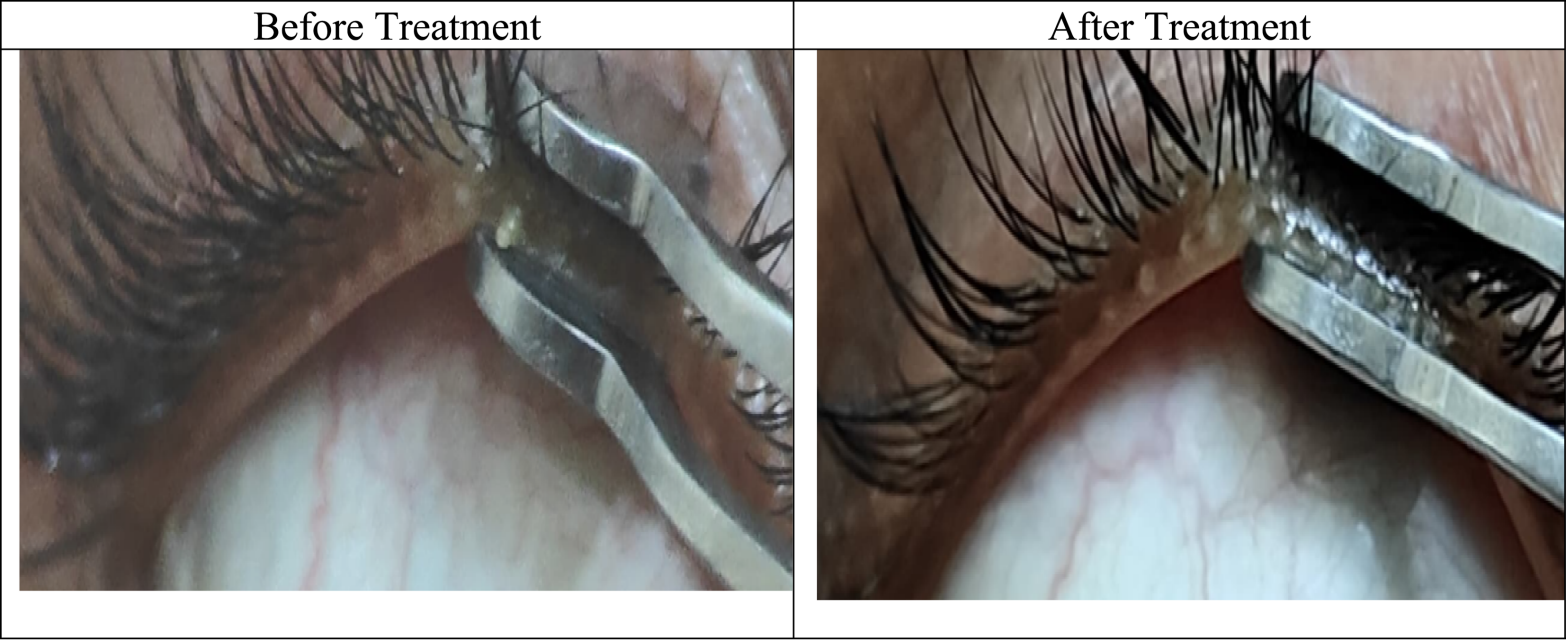
Before and after treatment photographs of a patient with MGD.

Following treatment, the median expressibility score improved to 3.0 (IQR 3.0–3.0) in both eyes and remained stable during both follow-up visits. As the post-treatment and follow-up values were identical for all participants, further inferential statistical testing was not applicable. The uniform improvement to the highest expressibility grade suggests effective relief of glandular blockage and restoration of meibum outflow (see Figure 4).

## Discussion

### Limitations of the study

This was an exploratory, single-arm clinical study with a modest sample size and a relatively short follow-up period. The lack of a control group limits direct comparison with standard or placebo treatments, and the use of patient-reported outcomes may have introduced some degree of subjective bias. Even so, the observations gathered offer meaningful preliminary evidence on the usefulness of Ayurvedic therapies in managing Meibomian Gland Dysfunction. These findings can serve as a foundation for future, larger-scale randomized trials aimed at confirming and extending the present results.

As this was a feasibility-oriented study without a control group, the findings are best interpreted as preliminary and exploratory rather than conclusive evidence of efficacy. The study design primarily aimed to document clinical observations, assess the practicality of the integrative protocol, and generate hypotheses for future research. Accordingly, the results should be viewed as early indicators that warrant validation through larger, controlled, and comparative trials.

*Patient experience and tolerability*: The combined intervention was well tolerated by all participants. No major adverse events such as burning, irritation, nasal regurgitation, or discontinuation due to discomfort were reported. Occasional transient watering occurred during initial applications and subsided without intervention.

#### Discussion on the effect of *shigrupallava arka*

*Shigrupallava*, the tender leaves of *Moringa oleifera* Lam, is traditionally described in Ayurveda as being useful in inflammatory ocular conditions, and contemporary experimental studies suggest that *Moringa oleifera* possesses anti-inflammatory, antioxidant, antimicrobial, and lipid-modulating properties (10–12). These properties provide a plausible biological basis for its inclusion in interventions targeting meibomian gland dysfunction, a condition characterized by glandular obstruction, inflammation, and altered lipid secretion.

In the present exploratory study, the use of *shigrupallava arka* as a topical ocular preparation was associated with improvements in clinical parameters related to gland patency and ocular surface stability. However, given the single-arm design and absence of a comparator group, these observations should be interpreted with caution. The findings do not establish efficacy but rather suggest a potential role for *Moringa oleifera* based formulations in supporting ocular surface health.

#### Discussion on the effect of *anu taila pratimarsha nasya*

*Pratimarsha nasya*, a form of daily nasal oil instillation described in Ayurveda (13), is traditionally considered supportive for conditions involving the head and sense organs (14). In the context of meibomian gland dysfunction, this intervention was included as a systemic supportive measure rather than a direct ocular treatment. The properties attributed to anu taila in classical texts suggest potential roles in maintaining mucosal hydration and supporting regional circulation, which may be relevant to ocular surface homeostasis.

In this exploratory study, the concurrent use of *pratimarsha nasya* with topical ocular therapies was associated with improvements in both subjective symptoms and objective ocular surface parameters. However, given the single-arm design, these observations should be interpreted cautiously. The contribution of nasal oil instillation cannot be isolated, and no conclusions regarding efficacy can be drawn. Instead, the findings suggest that *pratimarsha nasya* may have a complementary role within a broader integrative care approach.

#### Discussion on the effect of *avagundana*

*Avagundana*, a form of localized warm ocular fomentation traditionally described in Ayurvedic ophthalmic practice, was included in this study as a supportive local intervention for meibomian gland dysfunction. The procedure involves the application of gentle heat around the periocular region using a herbal poultice immersed in a decoction, which conceptually aligns with strategies aimed at relieving glandular obstruction and improving meibum flow.

In the present exploratory study, *avagundana* was used alongside other interventions during the initial phase of treatment and was associated with improvements in parameters related to gland expressibility and ocular surface comfort. From a contemporary perspective, this procedure is comparable to warm compress therapy commonly recommended in the management of meibomian gland dysfunction (15). However, given the multimodal treatment protocol and the absence of a control group, the specific contribution of *avagundana* cannot be isolated. The observations should therefore be interpreted as preliminary, suggesting a possible supportive role for localized warm fomentation with herbal preparations, which warrants further evaluation in controlled comparative studies.

The combined use of medicated eye instillation derived from *Moringa oleifera*, localized warm ocular fomentation with a herbal poultice, and daily nasal oil instillation represents an integrative Ayurvedic care approach aimed at addressing multiple aspects of meibomian gland dysfunction. Conceptually, the topical interventions target glandular obstruction and ocular surface disturbance, while the nasal oil instillation is intended as a supportive measure for lubrication and regional functional regulation.

In this exploratory study, the multimodal protocol was associated with improvements across several clinical parameters; however, the relative contribution of each component cannot be determined due to the combined administration and single-arm design. These observations should therefore be interpreted as preliminary. The findings suggest that an integrative, low-intensity approach addressing obstruction, surface inflammation, and tear film instability may warrant further evaluation in controlled studies to determine efficacy, durability of benefit, and optimal sequencing of interventions.

The treatment protocol was practical and feasible for primary-care settings. The procedures do not require specialized ophthalmic equipment, can be taught to patients or caregivers with minimal training, and can be administered safely in community clinics or at home. The medicines are inexpensive, easy to store, and have a long shelf life, which reduces cost and logistical burden. Time requirements were modest, with most daily applications taking less than 10–15 min. The main contraindications include acute ocular infection, recent ocular surgery, and hypersensitivity to any component of the formulation, which can be screened easily in outpatient practice. These features suggest that the protocol is well suited for implementation in primary care, particularly in resource-limited environments.

## Conclusion

This exploratory study suggests that a multimodal Ayurvedic care approach incorporating medicated eye instillation derived from *Moringa oleifera*, localized warm ocular fomentation, and daily nasal oil instillation may be associated with improvements in both patient-reported symptoms and objective ocular surface parameters in individuals with Meibomian Gland Dysfunction. The observed changes point toward potential benefits in tear film stability, gland expressibility, and ocular comfort; however, these findings should be interpreted cautiously given the single-arm design.

Conceptually, the combined protocol addresses multiple facets of MGD, including glandular obstruction, ocular surface disturbance, and lubrication support. While the concurrent administration of interventions limits attribution of effect to any single component, the overall observations provide preliminary signals that warrant further investigation. Larger, controlled studies with longer follow-up are required to determine efficacy, durability of benefit, and the specific contribution of each intervention within an integrative primary-care framework.

From a primary health care standpoint, such evidence-based traditional therapies offer a safe, affordable, and sustainable means of addressing common ocular conditions at the community level. Incorporating these approaches within integrated eye care models could enhance preventive strategies and help reduce the growing public health burden of dry eye and related disorders. Further large-scale, controlled, and mechanistic studies are encouraged to validate and extend these findings.

## Data Availability

The original contributions presented in the study are included in the article/supplementary material, further inquiries can be directed to the corresponding author.
